# Current situation of *Leishmania infantum *infection in shelter dogs in northern Spain

**DOI:** 10.1186/1756-3305-5-60

**Published:** 2012-03-27

**Authors:** Guadalupe Miró, Rocío Checa, Ana Montoya, Leticia Hernández, Diana Dado, Rosa Gálvez

**Affiliations:** 1 Departamento de Sanidad Animal, Facultad de Veterinaria, Universidad Complutense, Madrid, Spain

## Abstract

**Background:**

Canine leishmaniosis (CanL) caused by *Leishmania infantum *is a widespread endemic disease in the Mediterranean basin, though, so far, the north of Spain has been considered a non-endemic area. The aim of the present study was to determine the prevalence of specific antibodies to *L. infantum *among stray dogs living in shelters in this area, and to evaluate the clinical status (both clinical signs and clinico-pathological abnormalities) of seropositive dogs. Besides *L. infantum *infection, the epidemiological role of variables like sex, breed and age was also assessed.

**Methods:**

Over the year 2011 a cross-sectional study was conducted on a total of 418 stray dogs. A preliminary entomological survey was carried out using CDC-light traps. The chi-squared test was used to examine relationships between *L. infantum *seroprevalence and the remaining variables.

**Results:**

The overall seroprevalence of *L. infantum *infection detected was 3% in the Cantabrian coast. In Orense the seroprevalence was 35.6%. In this latter region, the presence of sand fly, *Phlebotomus perniciosus *was also detected.

In general, seropositivity for *L. infantum *was related to size (large breed dogs *versus *small) and age, with a significantly higher seroprevalence recorded in younger (0-3 years) and older dogs (> 7 years) than adult dogs. Clinical signs of CanL were observed in 41.3% of the seropositive dogs. The seropositivity for *L. infantum *infection associated with the presence of clinical signs and/or abnormal laboratory findings shows a prevalence of 4.5%.

**Conclusion:**

Our data provide new insight into the prevalence of CanL across northern Spain. The situation observed in Orense seems to be worsening compared to the few reports available, with figures being similar to those cited for known endemic areas of Spain. Besides, the presence of *P. perniciosus *in Orense points out to a risk of the spread of this zoonotic disease in this geographical area. These findings identify a need for an active search for the sand fly vectors of *L. infantum *across the entire northern spanish region including the rest of Galicia, Asturias, Cantabria and the Basque Country.

## Background

Canine leishmaniosis (CanL) is a widespread endemic disease in the Mediterranean basin. Its primary causative agent, *Leishmania infantum*, is transmitted to humans and animals by blood-sucking phlebotomine sand flies [1]. Dogs are the main domestic reservoir for human infection by *L. infantum *[2] .

A risk of the introduction of *L. infantum *infection in northern Europe is supported by the idea that changing factors linked to climate and environment could determine the expansion of the current geographical distribution range of the disease and its vectors [3-6]. The transport of infected dogs from endemic areas has also been attributed an important role in the spread of leishmaniosis towards the north of Europe [7-9]. In Germany, it has been estimated that some 20,000 infected dogs have been imported by tourists from endemic zones in southern Europe [7]. The presence of reservoirs and the detection of one of the parasite's main vectors, *Phlebotomus perniciosus*, in central European countries, such as Switzerland [10] or Germany [11], suggest the possible emergence of the disease at latitudes where it did not traditionally exist [12-14]. In effect, the distribution range of leishmaniosis across the European continent is no longer limited to the Mediterranean basin and, recently, new disease foci have been detected at the foothills of mountain ranges such as the Alps [15,16] or Pyrenees [17].

Estimates of canine *L. infantum *infection seroprevalence reported for Spain range from 3.7% for Orense province in the northwest corner of the country [18] to 34.6% for Málaga province on the south coast [19]. Northern Spain is presently considered a non-endemic area despite the sparse survey data available [18]. Recent works have reported the detection of CanL and its sand fly vectors, *Phlebotomus ariasi *and *P. perniciosus*, in a Pyrenean area of northeast Spain where the disease was previously unknown [20,21]. In Italy, the results of a recent study revealed the northward spread of CanL to a kennel in northern Italy, not previously affected by native infection foci [22]. Collectively, these observations suggest that knowledge of the real status of this disease in areas up until recently considered disease-free will help design control and prevention programmes with the final aim that veterinarians in these regions include CanL in their list of differential diagnosis tests.

The present study was designed to assess the status of *L. infantum *infection in northern Spain by examining dogs housed in six animal protection shelters across this large region. A preliminary sand fly survey was also conducted in the area of study.

## Methods

### Study area

The dogs examined were housed in kennels at six shelters, belonging to an animal protection organization, along the Cantabrian coast (northern Spain) and Orense. Climate and vegetation are typically oceanic, with warm summers and cool winters and rainfall evenly distributed all year round.

**Figure 1:**
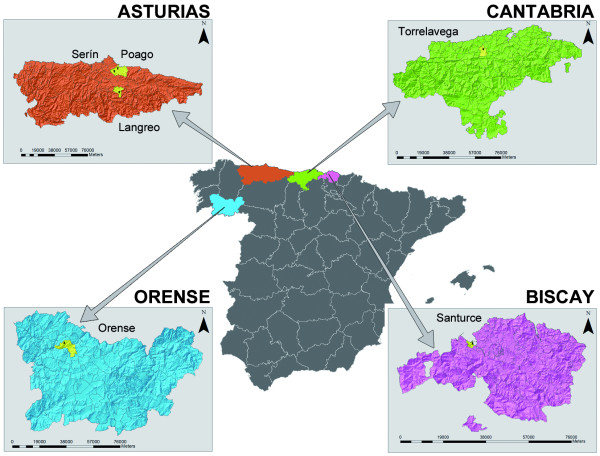
**Location of the study area in Spain**. Animal shelters surveyed shown on a digital elevation map: **Asturias **[Serín (43°30'14.17"N, 5°47'2.91"W), Poago (43°32'10.08"N, 5°44'20.69"W) and Langreo (43°17'46.90"N, 5°40'57.15"W)], **Cantabria **[Torrelavega (43°20'50.74"N, 4° 3'15.97"W)], **Orense **[Orense (42°22'0" N, 7°52'29"W)] and **Biscay **[Santurce (43°19'41.52"N, 3° 1'53.43"W)].

The shelters are located in Torrelavega (Cantabria); Gijón -Serín and Poago- and Langreo (Asturias), Orense (Galicia) and Santurce (Biscay) (Figure 1). These animal shelters are ideal for this type of study since they are situated in rural areas and the dogs live outdoors where they are exposed to the bites of hematophagous arthropods including phlebotomine sand flies. At these shelters, the animals were sterilized and maintained on a health control programme. The dogs were kept and handled according to the animal welfare standards upheld by Universidad Complutense de Madrid.

The characteristics of the six centres varied greatly. Thus, at the shelter in Torrelavega, some of the dogs were kept in individual huts with soil bedding and the rest were housed in groups of 3-5 dogs in covered open kennels without walls. At the shelters in Asturias (Gijón and Langreo) and Biscay (Santurce) all dogs were kept in groups of 4-5 dogs per kennel. Each kennel with a concrete floor had an indoor and outdoor area. In the animal shelter at Orense, the dogs were kept outside in yards (approximately 30-40 animals per yard) depending on the size and sex of the dogs.

### Data collection

Over the year 2011, our cross-sectional study was conducted on a total of 418 stray dogs from Torrelavega (Cantabria); Gijón -Serín and Poago- and Langreo (Asturias); Orense (Orense) and Santurce (Biscay) (Table 1).

**Table 1:** *Leishmania infantum *seroprevalence by locality

Locality	Total	Seropositivity (≥ 1:100) N (%)	Low titre (1/50) N (%)
Torrelavega	100	2 (2)	8 (8)
Serín, Poago and Langreo	171	8 (4.7)	7 (4.1)
Orense	101	36 (35.6)	23 (22.7)
Santurce	46	0 (0)	2 (4.3)

From each dog, a 5-6 ml blood sample was obtained by cephalic venipuncture. The collected blood was placed in four tubes containing: (1) lithium heparin (1 ml) for biochemical profile; (2) EDTA (0.5 ml) for full blood counts and blood smears; (3) EDTA (1 ml) to detect other CBVD (canine-borne vector diseases) by PCR; and (4) a tube without additives (2-3 ml) for antibody testing. The blood samples were kept at 4°C until processing and those mixed with EDTA for PCR were stored frozen at -80°C for a future epidemiological study. In addition, a clinical score was awarded to each dog in a thorough physical exam, evaluating both clinical signs and laboratory abnormalities (Table 2). We also took a picture of each animal and recorded the variables name, age, breed, sex, weight, and travelling history whenever possible.

**Table 2:** Scoring system categories based on clinical signs and clinic-pathological findings

CLINICAL SIGNS	0	1	2
Weight	normal	reduced	cachexia
Appetite	normal	reduced	anorexia
Behaviour	normal	apathy	postration
Lymphadenomegaly	absent	localized	generalized
Epistaxis	absent	moderate	severe
Keratoseborreic cutaneous lesions	absent	moderate	generalized
Ulcerative lesions	absent	simple	multiple
Onicogriphosis	absent	moderate	severe
Ocular lesions	absent	moderate	severe
Digestive disorders	absent	mild	severe
Arthropathy	absent	simple	multiple
Amyotrophy	absent	moderate	severe

CLINICO-PATHOLOGICAL FINDINGS	**0**	**1**	**2**

Plasma proteins	normal	elevated	reduced
A/G ratio	normal	reduced	-
Urea	normal	reduced	-
Creatinine	normal	elevated	-
ALT	normal	elevated	-

Complete blood count	0 = normal 1 = abnormal		

Clinical score: 0-35

### Laboratory tests

Serological testing for *L. infantum *consisted of detecting specific antibodies using the indirect immunofluorescence antibody test (IFAT) against in-house cultured promastigotes. Seroprevalence was calculated as the percentage of dogs testing positive for *L. infantum *antibodies. The serial dilutions prepared were ranged from 1/25 to 1/6400. The IFAT for anti-*Leishmania*-specific immunoglobulin G (IgG) antibodies was performed as described previously [23] using a cut-off ≥ 1:100 to denote seropositivity.

### Preliminary entomological survey

A preliminary entomological survey was carried out in September 2011 using CDC-light traps in Orense. Over two consecutive days, four CDC-light traps were set up in the afternoon and recovered early in the morning. Traps were placed no further than 3 m from the kennel. The animal shelter in Orense is near a small stream with riverside vegetation.

### Statistical analysis

Seropositivity frequencies were compared according to age, sex, and dog size (Table 3). The chi-squared test was used to examine relationships between *L. infantum *seroprevalence and the remaining variables. Differences were considered significant at p ≤ 0.05. All statistical tests were performed using SPSS 19.0 software (SPSS Inc., Chicago, IL, USA).

**Table 3:** *Leishmania infantum *seroprevalence by age, sex and size

	Total	Seropositive (N)	Seroprevalence (%)
**Age (years)**			
≤ 3	153	21	13.7
3 - 7	147	8	5.4*^(1)^
≥ 7	111	15	13.5

**Sex**			
Males	211	23	10.9
Females	204	23	11.3

**Size (kg)**			
Small (≤ 15)	97	5	5.2*^(2)^
Medium (15-25)	79	7	8.8
Large (≥ 25)	216	30	13.8

* p ≤ 0.05

(1) These differences were observed between adult and young dogs (p = 0.015) and between adult and older dogs (p = 0.024); (2) Significant differences were detected between small breed size compared to a large breed size (p = 0.023).

## Results

Over the year 2011, 418 stray dogs were examined in four regions of northern Spain. Using the cut-off of a *L. infantum *antibody titre ≥ 1:100 to denote seropositivity, seroprevalences for the different regions were: Cantabria 2% (2 out of 100), Asturias 4.7% (8 out of 171), Orense 35.6% (36 out of 101) and Biscay 0% (0 out of 47). Antibody titres recorded in seropositive animals ranged from 1/100 to 1/3200. Clinical signs compatible with CanL were observed in nineteen seropositive dogs, so the seroprevalence of *L. infantum *in clinically healthy and sick dogs was 58.7% (27/46) and 41.3% (19/46), respectively.

In general, antibody titres showed positive correlation with clinical scores, such that animals with higher clinical scores revealed higher antibody titres (≥ 1/400) (Table 4). Of note, among the animals exhibiting low antibody titres (along with low clinical scores), dog OR100 (see Table 4) had a high clinical score suggesting some other underlying disease or coexisting infection.

**Table 4:** Clinical scores recorded in the seropositive dogs

DOG NUMBER	IFAT *Leishmania*	CLINICAL SCORING
OR 4	1/100	0
OR 8	1/800	4
OR 10	1/1600	11
OR 19	1/100	0
OR 22	1/200	1
OR 30	1/200	0
OR 43	1/800	0
OR 44	1/200	2
OR 46	1/3200	6
OR 47	1/200	4
OR 48	1/100	0
OR 51	1/100	0
OR 52	1/200	2
OR 54	1/200	4
OR 56	1/400	0
OR 57	1/200	2
OR 60	1/200	0
OR 62	1/200	5
OR 65	1/400	0
OR 67	1/200	2
OR 69	1/200	5
OR 70	1/400	0
OR 72	1/200	0
OR 74	1/400	6
OR 77	1/800	0
OR 80	1/200	0
OR 83	1/3200	3
OR 84	1/800	6
OR 85	1/400	0
OR 88	1/200	0
OR 94	1/400	15
OR 95	1/200	2
OR 98	1/200	4
OR 99	1/100	0
OR 100	1/100	9
OR 101	1/100	0
AS 18	1/100	0
AS 72	1/100	0
AS 100	1/100	3
AS 139	1/100	0
AS 141	1/100	0
AS 142	1/100	0
AS 152	1/100	2
AS 162	1/100	0
SA 36	1/100	0
SA 61	1/100	2

*OR = Orense; AS = Asturias; SA = Santander (Cantabria)

The age of the dogs ranged from 4 months to 13 years. When the dogs were stratified into three categories: young (< 3 years), adult (3 ≤ years < 7) and older dogs (≥ 7 years), significant differences were observed in *L. infantum *seroprevalence (*p *= 0.036). These differences were observed both between adult and young dogs (*p *= 0.015) and between adult and older dogs (*p *= 0.024), the adult group differing significantly from the other two groups. Hence, age exhibited a bimodal distribution of seroprevalence with a peak in young dogs and a second larger peak observed in the older dogs.

Significant differences in CanL infection risk were detected by breed according to size (*p *= 0.023). Thus, a small breed size was a protective factor compared to a large breed size. In addition, the seroprevalence recorded in females (10.9%) was slightly lower than in males (11.3%)and not significant (*p *= 0.903).

In our preliminary study conducted in Orense, eight sand flies were collected using CDC-light traps. All were identified as *P. perniciosus*. One male and one female were captured on September 26^th ^2011, and four males and two females on September 27^th ^2011.

## Discussion

The seroprevalence of *L. infantum *in Spain varies across a geographical gradient (3.7 - 34.6%), with highest prevalences cited for southern and eastern Spain (Mediterranean coast) [19]. Despite the detection of CanL in a Pyrenean area of northeast Spain where the disease was previously unknown [20,21], its substantially low prevalence in the north of the country (3.7-4.4%) has earned the consideration of this region so far as a low endemic area [18]. Similar seroprevalences were recorded in the present study performed in Asturias, Biscay and Cantabria (0-4.7%), in which the presence of *L. infantum *was detected in two out off three shelters of sampled dogs. On the other hand, the seroprevalence observed in Orense (35.6%) constitutes a considerable increase over this latest reported estimate [18] similar to the figures quoted for Spanish areas classed as endemic. Amusategui et al. (2004) detected a similarly high seroprevalence (29.2%) of *L. infantum *in Valdeorras, a town close to our Orense sampling site. Probably, the current bioclimatic characteristics of this geographic area are compatible with the presence and transmission of *L. infantum *according to the prediction models described for other regions in Europe [24-26]. Effectively, the preliminary data obtained in the present study demonstrate the presence of *P. perniciosus *in the area of the animal shelter in Orense. Also *P. perniciosus *was found by other authors in Orense [27]. Moreover, *P. ariasi *was found in Galicia (Lugo and Orense) [28,29]. We were unable to address the presence of sand flies at the other animal shelters since the weather conditions were unsuitable for sand flies.

The dogs included in our study were 4 months to 13 years of age. *L. infantum *seroprevalence was higher in dogs aged 0-3 years (13.7%) or older than 7 years (13.5%) than in those 3-7 years old (5.4%). Thus, seroprevalence showed a bimodal distribution with a peak in dogs under 3 years and another peak between the ages of 8 and 10 years. This pattern suggests that *L. infantum *infection is dependent on host immunological status [30,31]. Similar data have been reported in a study conducted in Madrid, Spain [32], while other authors have only noted a higher prevalence in older dogs suggesting that susceptibility to *L. infantum *infection increase with age [31,33-35].

In our study, dog size emerged as an epidemiological variable related to *L. infantum *infection such that seroprevalence was significantly higher in the large breed dogs (13.8%) compared to the small breed dogs (5.2%), consistent with the results of others [32,36]. A possible explanation could be that the greater body mass of larger dogs determines a larger surface area exposed to bites of the arthropod vector, as suggested for other vector borne disease such as canine thelaziosis [37]. Alternatively, it could be that medium or large-sized dogs are those most used for farm activities requiring they remain outdoors for long periods of time [32].

Although in some cases a higher prevalence of leishmaniosis has been observed among male dogs [38], we detected no such link between *L. infantum *infection and sex (seroprevalence 10.9% in males versus 11.3% in females), as mentioned previously by other authors [18,32,35,39].

Nineteen out of 46 seropositive dogs (41.3%) showed clinical signs of CanL, mainly enlarged lymph nodes (13/46), skin lesions (17/46) and weight loss (14/46). However, the prevalence of *L. infantum *infection was higher in the clinically healthy dogs (58.7%) than in those showing some of these signs (41.3%). Similar observations have been made by others [33,35,40]. The high percentage of subclinical infection may suggest some level of cellular immune response developing over time, which is believed to limit disease outcome [41], or that these dogs were still in the early stages of disease since many clinically healthy seropositive dogs had low antibody titres (1/100). Thus, in Cantabria and Asturias all seropositive dogs had a titre of 1/100 whilst in Orense only 7 of the 36 seropositive dogs had titres as low as 1/100. In these particular dogs along with dogs showing 1/50 titres (see Tables 1 and 4), we will, in the early future, be determining subsequent antibody changes to assess their seroconversion and clinical status.

We should highlight that over 25% (13 out of 46 had no clinical signs nor abnormal laboratory findings) of the seropositive dogs were clinically healthy with titres of 1/100. These dogs could pose a risk of infection aggravated by their non-ideal living conditions (affected by stress or concomittant diseases). It is especially important that this type of stray animal be monitored both in endemic and non endemic areas.

## Conclusions

In conclusion, our findings reveal a low seroprevalence of *L. infantum *infection in stray dogs along the Cantabrian coast, on the contrary in the Orense province the prevalence was similar to that reported in endemic areas of Spain. The concurrent detection of *P. perniciosus *in Orense suggests a risk of the spread of this zoonotic disease, and prompts further studies designed to actively search for the sand fly vectors of CanL in nearby areas of Asturias, Cantabria and Biscay.

## Competing interests

The authors declare that they have no competing interests.

## Authors' contributions

GM designed the survey, drafted the first version of the manuscript and finalized the manuscript. RC, AM, LH and DD carried out the field survey and laboratory work and finalized the manuscript. RG performed the entomological study, drafted the first version of the manuscript and finalized the manuscript. AM, RG and RC performed the statistical analysis of data. All the authors reviewed the manuscript.
